# Methylenedioxymethamphetamine (MDMA)-Induced Hyponatremia: Case Report and Literature Review

**DOI:** 10.7759/cureus.15223

**Published:** 2021-05-25

**Authors:** Sherif Elkattawy, Ahmed Mowafy, Islam Younes, Marina Tucktuck, James Agresti

**Affiliations:** 1 Internal Medicine, Rutgers New Jersey Medical School/Trinitas Regional Medical Center, Elizabeth, USA; 2 Internal Medicine, St. George's University School of Medicine, True Blue, GRD; 3 Nephrology, Trinitas Regional Medical Center, Elizabeth, USA

**Keywords:** mdma, ecstasy, hyponatremia, methylenedioxymethamphetamine, recreational drug

## Abstract

3,4-Methylenedioxymethamphetamine, MDMA, or “ecstasy”, is a trending recreational drug used by the young crowd for obtaining "euphoria." Over the past few years, there have been multiple reports of teenagers committing suicide and suddenly dying post ingesting MDMA. Compared to other illicit drugs such as heroin, hash and cocaine, ecstasy is relatively new hence the popularity. There are multiple toxicities associated with MDMA, including but not limited to seizures, depression, liver failure, or thrombosis. However, in this report, we will focus on hyponatremia and one of the most feared complications of such electrolyte disturbance: seizures. The rapid reversal of the hyponatremia with hypertonic saline in such acute setting is key to reduce risk of cerebral swelling. We report a case of a young female with no past medical history who presented to emergency department post ecstasy use with tonic-clonic seizure and hyponatremia.

## Introduction

3,4-Methylenedioxymethamphetamine (MDMA, or “ecstasy”) is a common recreational drug. MDMA has potent sympathomimetic and serotonergic effects. The serotonergic actions of MDMA are the presumed mechanism of euphoria and feeling of happiness among its users [1]. MDMA has been associated with acute and chronic toxicities. Acute toxicities of MDMA include agitation, hyperthermia, acute liver toxicity, fulminant liver failure, rhabdomyolysis, and disseminated intravascular thrombosis [2,3]. Chronic use of MDMA was found to be associated with depression, neurotoxicity, and impaired cognitive function [4]. Hyponatremia has been also reported with MDMA use, however, the pathophysiology is not completely understood. MDMA-induced hyponatremia may be acute and severe enough to cause seizures, coma, and even death secondary to cerebral edema [3].

## Case presentation

**Figure 1:**
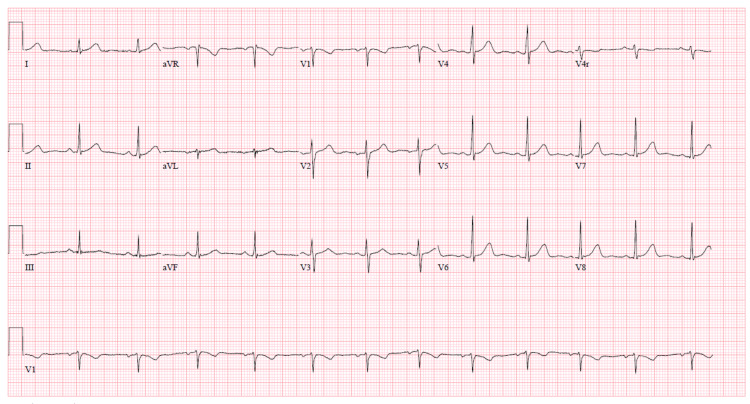
Electrocardiogram (EKG) significant for normal sinus rhythm with no QTC prolongation

**Figure 2:**
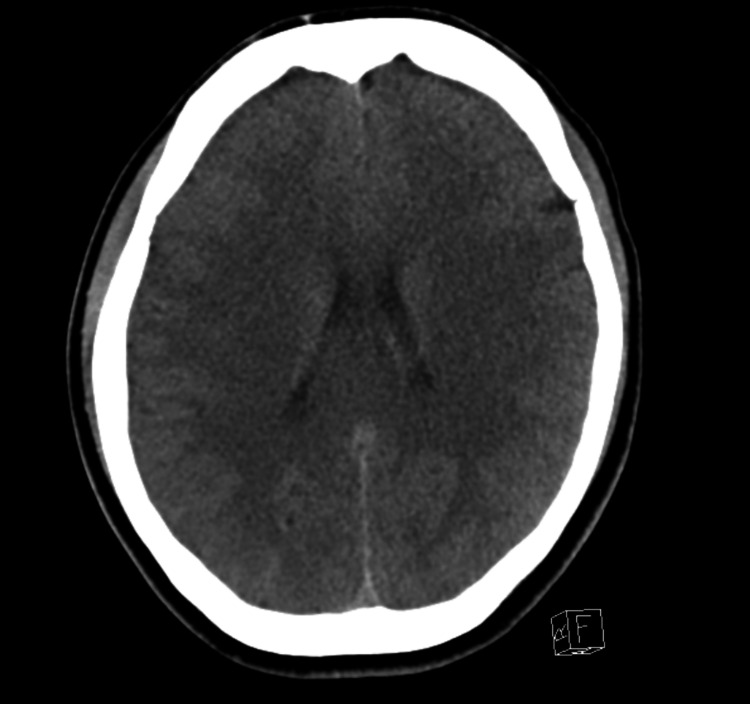
CT scan of the head was negative for bleeding or infarction

A 29-year-old female patient with no past medical history presented to the ED after experiencing a witnessed episode of generalized, tonic-clonic seizures. Prior to presentation, the patient had ingested an unknown quantity of MDMA (ecstasy), after which she had an episode of generalized tonic-clonic seizures associated with loss of consciousness, but no vomiting, urinary/fecal incontinence. The patient had no prior history of seizure disorder. On presentation, the patient was postictal, afebrile, vitally stable [Heart Rate (HR) 98, Respiratory Rate (RR) 18, Blood Pressure (BP) 116/63]. Physical examination was non-pertinent. Blood chemistry revealed a hemoglobin (Hgb) of 12.8 gm/dL (12-16 gm/dL), white blood cell (WBC) 15.8 (4.8-10.8), with absolute neutrophil count of 12.9 (1.4-6.5) and lymphocytes 2.3 (1.2-3.4), serum sodium 117 mMol/L (136-146 mMol/L), potassium 5.1 mMol/L (3.6-5.1 mMol/L), chloride 88 mMol/L (101-111 mMol/L), bicarbonate 17 mMol/L (22-32 mMol/L), creatinine 0.72 mg/dL (0.4-1.0 mg/dL), blood urea nitrogen (BUN) 7 mg/dL (8-20 mg/dL), creatine phosphokinase (CPK) 1123 U/L (26-140 U/L). Urine analysis was positive for amphetamines, and revealed urine osmolarity 248 mOsm/kg, urine sodium 36 mMol/L, urine potassium 165 mMol/L, urine chloride 54 mMol/L. Serum alcohol, salicylate, tylenol levels were negative; thyroid stimulating hormone (TSH) 2.1 mIU/mL (0.34-5.6 mIU/mL), vitamin B12 435 pg/mL (180-914 pg/mL). Electrocardiogram (EKG) showed normal sinus rhythm with no QTC prolongation as seen in Figure 1. CT of the head was negative for bleeding or infarction as shown in Figure 2.

The patient was given 500cc of normal saline and started on hypertonic saline to correct the hyponatremia. Serum sodium increased to 129 mMol/L in the following two hours, so the IV fluids were changed to Dextrose 5%, but the sodium continued to climb, reaching 134 mMol/L after 3 hours from presentation. The patient was given two doses of 1 mcg of Vasopressin, reducing serum sodium to 130 mMol/L. The patient’s mental status improved, and she returned to a fully oriented state in 8 hours. Neurological examination was negative for motor or sensory deficits, deep tendon reflexes were within normal limits. Serum sodium remained stable around 128-130 mMol/L throughout the rest of her hospital stay and she was discharged with extensive counseling on avoiding illicit drug use.

## Discussion

MDMA or ecstasy is a synthetic sympathomimetic amphetamine that traditionally had widespread use among teenagers at “rave” parties [5-7]. However, since 2013, MDMA use has witnessed a rise among young adults, and females between ages 15-30 years are disproportionately affected [5,8,9]. Its falsely “safe” drug profile coupled with misconception about water intake after ingestion has left many unaware of its toxic and fatal complications, mainly hyponatremia and seizures leading to increased emergency admissions and mortality [9].

MDMA exerts its actions on serotonin, dopamine and epinephrine by increasing their presence in the synaptic cleft and inhibiting their reuptake [5,7,10,11]. The effects of MDMA on the body encompass its action as an amphetamine derivative as well as exhibiting serotonergic toxicity [7,11]. This mechanism leads to increased sympathetic activity, which accounts for the drug’s euphoria, reduced fatigue/increased energy, increased sociability, and increased alertness and mental powers [5,7,10]; these effects are thought to only last for a few hours. Its use has also been associated with transient increases in body temperature, dry mouth, increased thirst, hot flashes, sweating and tachycardia due to central nervous system (CNS) stimulation [10]. It is believed that the release of serotonin affects the body’s temperature regulation leading to the hyperthermia that contributes to excessive thirst among users [11]. In return, it is thought that the polydipsia leads to the hyponatremia and increased secretion of antidiuretic hormone (ADH) [5,11]. In addition, one of the most prominent effects of MDMA on the body is the renal toxicity, which manifests through acute kidney injury (i.e., non-traumatic rhabdomyolysis) or water and electrolyte imbalance, clinically seen as hyponatremia [5,12]. The resulting hyponatremia also paves way to seizures through increased CNS stimulation, cerebral edema and increasing intracranial pressure which can also trigger seizures [11,13].

MDMA-induced hyponatremia, usually <120 mMol/L, is one of the most serious complications arising from ingestion of even a single dose of MDMA, which is believed to arise several hours after use [3,5]. There seems to be a consensus among studies that there are two plausible theories for the mechanism of action. The first one is that the hyponatremia occurs through syndrome of inappropriate anti-diuretic hormone production (SIADH) and water intoxication [13,14]. Specifically, MDMA’s structural resemblance to serotonin and MDMA metabolites work to increase the concentration of serotonin and dopamine [5,11,14-17]. These neurotransmitters act on increasing the release of arginine vasopressin (AVP) in the brain leading to SIADH (especially inappropriate elevation in urine osmolarity from 184 to 970 mOsm/Kg) and increasing water retention by the body [3,5,10,11,13]. The second theory is the increased availability of fluids and excessive water intake due to the hyperpyrexia following MDMA ingestion as well as MDMA’s effect on causing dry mouth [3,10,11]. As for MDMA’s heightened effect on females compared to males, research has speculated that estrogen decreases the Na-K-ATPase pump inhibiting sodium release from the brain as well as findings that AVP’s vasoconstrictive effects are more pronounced in female brains compared to males [5,6,10,15].

Our patient’s sodium levels upon admission were 117 mMol/L, which is consistent with the sodium levels (<120) for MDMA-induced hyponatremia. She also had a CPK of 1123 U/L, suggestive of non-traumatic rhabdomyolysis, a complication also seen in other studies [5,10,12]. However, our patient did not have other common symptoms, such as cardiac tachyarrhythmias, which has been reported in other studies [5,10-12].

The management and approach to patients suspected of MDMA-induced hyponatremia depend on the severity of the symptoms and require acting in a timely fashion to prevent the progress of hyponatremia complications. In patients with mild to moderate symptomatic hyponatremia, fluid restriction and observation of water-balance spontaneous correction seems to be the recommended course of action [3,5,11]. In those with severe hyponatremia, it is agreed that 0.9% NaCl or hypotonic solutions should be avoided, especially among patients with excessive water retention and elevated urine osmolarity [5]. Instead, hypertonic 3% saline fluid should be administered [3,5,11,14]. One study has suggested mannitol as an osmotic diuresis as a treatment for symptomatic MDMA-induced hyponatremia [12]. The ongoing management of MDMA-induced hyponatremia should also include correction of the electrolytes, monitoring urine output, urine osmolarity and serum levels of sodium. Body temperature should also be monitored to decrease MDMA-associated hyperpyrexia [5]. In addition, education about the misconception on water intake following MDMA ingestion and the serious and fatal complications should be part of the comprehensive approach to MDMA-induced hyponatremia. Our patient’s sodium level progressively corrected from 117 to a range of 128-130 mMol/L with a careful treatment plan involving hypertonic solution as well as vasopressin.

MDMA ingestion should be suspected in any young female presenting to the emergency department with a new-onset seizure and/or hyponatremia. Our case was an example of MDMA-induced hyponatremia among a female patient post ingestion of an unknown dose. Our approach and management of MDMA-induced hyponatremia is in sync with the proposed recommendations. Careful awareness and monitoring of the sequence of hyponatremia-related complications is thus warranted.

## Conclusions

MDMA toxicity should be considered in the differential diagnosis of patients presenting to the emergency department with unexplained coma and hyponatremia. MDMA-induced hyponatremia can be severe enough to cause morbidity and mortality. Early diagnosis and proper treatment with close monitoring of electrolytes are vital for a better outcome.
